# Thickness-dependent electro–chemo–mechanical modelling of SnS_2_/graphene anodes for high-performance potassium-ion batteries

**DOI:** 10.1039/d6ra00114a

**Published:** 2026-03-19

**Authors:** Ghada Al-Assi, Ahmed Basheer Ayyed, Roopashree R, Subhashree Ray, Baraa Mohammed Yaseen, Kavitha V, Renu Sharma, Aashna Sinha, Milad Safamanesh

**Affiliations:** a Faculty of Allied Medical Sciences, Hourani Center for Applied Scientific Research, Al-Ahliyya Amman University Amman Jordan; b College of Dental Medicine, Department of Dental Medicine, Al-Turath University Baghdad Iraq; c Department of Chemistry and Biochemistry, School of Sciences, JAIN (Deemed to be University) Bangalore Karnataka India; d Department of Biochemistry, IMS and SUM Hospital, Siksha ‘O’ Anusandhan Bhubaneswar Odisha 751003 India; e Department of Medical Laboratory Technics, College of Health and Medical Technology, Alnoor University Mosul Iraq; f Department of Chemistry, Sathyabama Institute of Science and Technology Chennai Tamil Nadu India; g Department of Chemistry, University Institute of Sciences, Chandigarh University Mohali Punjab India; h School of Applied and Life Sciences, Division of Research and Innovation, Uttaranchal University Dehradun Uttarakhand India; i Young Researchers and Elite Club, Islamic Azad University Tehran Iran miladsafamanesh.academic@gmail.com

## Abstract

Potassium-ion batteries (KIBs) have emerged as a promising alternative to lithium-ion systems; nevertheless, their large-scale application is critically constrained by the coupled transport and mechanical degradation of high-capacity alloy-type anodes. In this study, a fully coupled electro–chemo–mechanical finite-element multiphysics framework is established to systematically investigate thickness-dependent transport behavior, stress evolution, and capacity degradation in SnS_2_/graphene nanocomposite anodes. Distinct from conventional electrochemical-only models, the proposed approach incorporates genuine bidirectional coupling, whereby potassium-ion concentration induces chemical strain and mechanical stress, while stress evolution dynamically modulates ionic diffusivity and reversible capacity. Numerical simulations performed over a wide electrode thickness range (5–40 µm) reveal a clear transition from diffusion-dominated behavior in thin electrodes to stress-limited operation in thicker, practically relevant configurations. Thin electrodes (∼5 µm) exhibit nearly homogeneous potassium distribution, low peak stresses (∼0.27 GPa), and negligible capacity decay, whereas thick electrodes (∼40 µm) develop severe concentration gradients, elevated stresses approaching ∼0.7 GPa, and accelerated stress-driven capacity fade. Notably, an optimal intermediate thickness of approximately 10 µm is identified, achieving a favorable balance between transport efficiency and mechanical stability with capacity retention exceeding 90% upon cycling. Quantitative agreement with experimental data for sub-5 nm SnS_2_/graphene anodes confirms the predictive capability of the model. This work provides mechanistic insight and practical design guidelines for the scalable development of mechanically robust, high-performance potassium-ion battery anodes.

## Abstract 2

A coupled electro–chemo–mechanical model reveals thickness-dependent transport and stress effects in SnS_2_/graphene anodes for potassium-ion batteries.

## Graphical abstract

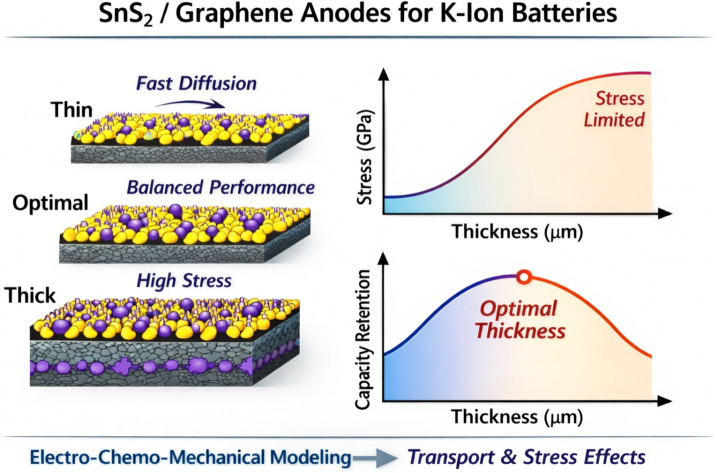

## 1. Introduction

Potassium-ion batteries (KIBs) have emerged as a promising alternative to lithium-ion batteries owing to the natural abundance, low cost, and favorable redox potential of potassium. These attributes render KIBs particularly attractive for large-scale energy storage applications where economic and resource sustainability is critical.^1–3^ However, the practical implementation of KIBs remains fundamentally constrained by the lack of suitable anode materials that can simultaneously deliver high capacity, fast kinetics, and long-term mechanical stability. Compared to lithium ions, the substantially larger ionic radius of K^+^ induces severe volume expansion, sluggish solid-state diffusion, and accelerated structural degradation, posing formidable challenges for electrode design and durability.^4–7^

Among the various anode candidates explored to date, tin-based chalcogenides (especially SnS_2_) have attracted considerable attention due to their high theoretical capacity derived from multi-electron conversion and alloying reactions with potassium.^8,9^ Nevertheless, bulk SnS_2_ suffers from dramatic volume changes exceeding 200% during potassiation and depotassiation, which lead to particle pulverization, loss of electrical contact, and rapid capacity decay.^10,11^ Embedding sub-5 nm SnS_2_ nanoparticles within conductive graphene frameworks is expected to alleviate thickness-induced transport and mechanical limitations by reducing diffusion lengths and buffering volumetric expansion, as systematically analyzed in this work. While such nanocomposite architectures exhibit excellent electrochemical performance at laboratory scale, translating these gains to practical electrode thicknesses remains a critical and unresolved challenge.^12–15^

In realistic battery electrodes, thickness plays a decisive role in governing ion transport, stress evolution, and degradation behavior.^16^ Increasing electrode thickness is essential for achieving commercially relevant areal capacities, yet it inevitably amplifies potassium-ion concentration gradients, induces heterogeneous volumetric expansion, and generates large internal stresses under mechanical constraint.^17,18^ Despite growing recognition of these coupled phenomena, most existing modeling studies of KIB anodes focus predominantly on electrochemical transport and reaction kinetics, treating mechanical effects as secondary or neglecting them altogether. Conversely, mechanical models often assume uniform ion distributions and fail to capture stress feedback on electrochemical processes. This decoupled treatment obscures the mechanistic origins of thickness-dependent performance degradation and limits predictive capability.^19–21^

To address these deficiencies, fully coupled electro–chemo–mechanical multiphysics modeling has gained increasing attention in lithium-ion battery research as an effective tool for resolving the complex interactions between ion transport, electrochemical reactions, and mechanically induced degradation.^22,23^ Such frameworks have demonstrated that mechanical deformation and stress localization can substantially influence reaction distribution, transport kinetics, and electrode utilization, particularly in high-capacity alloy-type systems.^22,23^ Finite-element-based multiphysics platforms, such as COMSOL Multiphysics, have enabled the numerical integration of electrochemical transport equations with solid mechanics, offering spatially resolved insight into concentration gradients, chemical strain, and stress evolution across porous electrodes. Nevertheless, the majority of these models remain focused on lithium-based chemistries, often at limited length scales or under simplified assumptions that neglect thickness-dependent effects and realistic electrode constraints.^24,25^

In potassium-ion battery systems, and especially for high-capacity alloying anodes such as SnS_2_-based nanocomposites, comparable fully coupled multiphysics frameworks remain scarce. Existing models for KIBs largely emphasize electrochemical performance while treating mechanical effects in a unidirectional manner, where stress is merely a passive outcome of ion insertion.^26^ Crucially, bidirectional coupling (whereby evolving mechanical stress actively modulates ionic diffusivity, reaction kinetics, and reversible capacity over cycling) is rarely incorporated. As a consequence, the fundamental transition from diffusion-dominated behavior in thin electrodes to stress-limited operation in thick, practically relevant electrodes is still poorly understood. This gap restricts the predictive design of KIB electrodes and underscores the need for a rigorously coupled electro–chemo–mechanical modeling approach capable of capturing stress feedback, thickness scaling, and long-term degradation within a unified multiphysics framework.^27–29^

In this work, we develop a fully coupled electro–chemo–mechanical multiphysics finite-element model to systematically investigate potassium-ion transport, chemically induced deformation, stress evolution, and capacity fade in SnS_2_/graphene nanocomposite anodes over a broad range of electrode thicknesses. The framework is implemented in COMSOL Multiphysics by integrating electrochemical transport and reaction kinetics with solid-mechanics-based deformation, enabling spatially and temporally resolved analysis of coupled fields under realistic galvanostatic operation. By explicitly linking local K^+^ concentration to chemical strain and incorporating stress-dependent ionic diffusivity together with mechanically driven capacity degradation, the model captures the essential bidirectional interactions that govern electrode performance beyond electrochemical-only descriptions. Calibrated and validated against experimental data for sub-5 nm SnS_2_/graphene anodes, the model enables quantitative identification of thickness-dependent operating regimes, reveals optimal electrode thickness ranges, and elucidates the mechanistic origins of stress-induced performance degradation. Collectively, this study provides both fundamental insight and practical design guidelines for the scalable development of durable, high-capacity potassium-ion battery anodes beyond the laboratory scale.

## 2. Methodology

This section describes the development and implementation of a fully coupled electro–chemo–mechanical multiphysics model for SnS_2_/graphene nanocomposite anodes in potassium-ion batteries (KIBs). The framework integrates electrochemical transport, reaction kinetics, and mechanical deformation to capture bidirectional interactions between K^+^ insertion/extraction, volumetric strain, and stress evolution across varying electrode thicknesses (5–40 µm). Simulations were performed using COMSOL Multiphysics (version 6.2), coupling the transport of diluted species, electrochemistry, and solid mechanics modules. Model parameters were calibrated from experimental data on sub-5 nm SnS_2_ nanoparticles embedded in graphene. This approach enables quantitative prediction of thickness-dependent degradation mechanisms, addressing limitations in prior models that often neglect mechanical feedback or scale-up effects.

The primary innovation of this multiphysics model is its bidirectional electro–chemo–mechanical coupling, explicitly linking K^+^ transport, chemical strain, and stress feedback, while systematically varying electrode thickness (5–40 µm) to bridge laboratory and practical scales. Key advancements include: quantitative thickness-dependent analysis revealing optimal (∼10 µm) and stress-limited (∼40 µm) regimes; stress-modulated diffusivity for mechanical transport inhibition; a critical threshold (*c*_K_ < 0.7 *c*_K,max_) to prevent yielding; and prediction of accumulated stress-driven capacity fade.

The detailed theoretical framework, governing equations, numerical implementation, and parameter sources used in the simulations are provided in the SI (Computational methods section).

### 2.1. Geometry and parameters

The computational domain adopts a representative volume element (RVE) strategy to efficiently capture the multiscale microstructure of the SnS_2_/graphene nanocomposite anode while accommodating electrode thicknesses ranging from 5 µm (laboratory-scale, diffusion-dominated) to 40 µm (near-industrial, stress-limited). The RVE comprises sub-5 nm SnS_2_ nanoparticles (average diameter ∼3.8 nm, 76.8 wt% loading) uniformly embedded within a porous graphene matrix, consistent with experimental observations.^30^ Electrode thickness is explicitly varied as a parametric sweep, with the aluminum current collector represented as a fixed boundary to emulate high adhesion strength. Key simulation parameters, extracted and calibrated from multiple scientific sources,^31–34^ are summarized in Table 1. These values ensure physical realism across electrochemical, transport, and mechanical domains.

**Table 1:** Key simulation parameters, sources, and calibration ranges^30–34^

Parameter	Symbol/value (range)	Physical meaning	Source/references
SnS_2_ nanoparticle diameter	∼3.8 nm (sub-5 nm)	Active particle size	Bin *et al.*^30^
SnS_2_ weight fraction	76.8 wt%	Active material loading	Bin *et al.*^30^
Electrode porosity	0.45 (0.40–0.50)	Void fraction	Bin *et al.*,^30^ Chen *et al.*^17^
Effective diffusion coefficient	DK = 2 × 10^−13^ m^2^ s^−1^ (1 to 5 × 10^−13^)	K^+^ diffusivity	Zhang *et al.*,^37^ calibrated
Chemical expansion coefficient	*β* = 0.45 (0.40–0.50)	Volumetric strain factor	Christensen & Newman,^31^ Zhao *et al.*^32^
Composite Young's modulus	*E* = 320 GPa (250–400 GPa)	Elastic stiffness	Zhao *et al.*,^32^ Bucci *et al.*^33^
Stress–diffusion coupling	*λ* = 1 × 10^−9^ Pa^−1^ (0.5 to 2 × 10^−9^)	Stress inhibition factor	Luo *et al.*,^39^ fitted
Stress-fade coefficient	*γ* = 5 × 10^−4^ GPa^−1^ s^−1^ (3 to 7 × 10^−4^)	Mechanical degradation rate	Bin *et al.*,^30^ calibrated
Specific surface area	*a*_s_ = 78 m^2^ g^−1^	Reaction interface area	Bin *et al.*^30^
Exchange current density	*j*_0_ = 10^−3^ A m^−2^	Charge transfer kinetics	Bin *et al.*,^30^ EIS fitting
Operating temperature	*T* = 298 K	System temperature	Experimental condition^30^

Parameter ranges reflect uncertainties and sensitivities derived from experimental variability (*e.g.*, particle size distribution) and literature on related alloy systems.^17,35^ Graphene's high in-plane stiffness and conductivity provide essential buffering, while porosity accommodates expansion. This parameterized framework enables systematic exploration of thickness effects without altering intrinsic material properties, ensuring predictive fidelity across scales.

#### 2.1.1. Parameter selection, calibration, and modeling assumptions

All physical, electrochemical, and mechanical parameters used in the present simulations were obtained from a combination of experimental literature, established theoretical models, and targeted numerical calibration. Table 1 summarizes the adopted values, corresponding uncertainty ranges, and primary literature sources.

Structural parameters, including nanoparticle size, weight fraction, porosity, and specific surface area, were directly extracted from the experimental characterization of SnS_2_/graphene composites reported by Bin *et al.*^30^ These parameters define the representative microstructure used in the RVE domain. Transport and kinetic parameters, such as the potassium diffusion coefficient and exchange current density, were initially estimated from reported galvanostatic intermittent titration (GITT), electrochemical impedance spectroscopy (EIS), and rate capability measurements in SnS_2_-based systems.^30,37^ These values were subsequently refined through calibration against experimental capacity and polarization data to ensure quantitative agreement.

Mechanical properties, including Young's modulus and chemical expansion coefficient, were derived from analogous alloy-type electrode studies and continuum mechanics models.^31–33^ In the absence of direct mechanical characterization for potassiated SnS_2_, these parameters were adjusted within physically reasonable bounds to reproduce experimentally observed volume expansion and stress levels. Coupling coefficients (*λ* and *γ*), which govern stress-modulated transport and stress-driven degradation, were determined through parametric sensitivity analysis and fitted to reproduce reported cycling stability and capacity fade trends.^30,39^

Several simplifying assumptions were adopted to ensure computational efficiency and numerical stability. The composite electrode was treated as an effective homogeneous continuum at the RVE scale, and chemical expansion of SnS_2_ nanoparticles was assumed to be isotropic. All simulations were performed under isothermal conditions at 298 K, while electrolyte depletion, side reactions, and SEI formation were neglected. In addition, interfacial contact resistance was assumed to remain constant throughout cycling. These assumptions allow efficient resolution of the coupled electro–chemo–mechanical fields while retaining the dominant mechanisms governing thickness-dependent behavior.

Parameter uncertainty ranges reported in Table 1 reflect experimental variability and literature dispersion. Sensitivity analyses confirmed that the main conclusions regarding thickness-dependent regimes remain robust within these bounds. These modeling choices and documented parameter sources ensure that the proposed framework is transparent, reproducible, and suitable for independent verification.

### 2.2. Governing equations

The electrochemical behavior of the SnS_2_/graphene nanocomposite anode is described by coupled governing equations for ion transport and reaction kinetics, implemented within a porous electrode framework.

#### 2.2.1. Electrochemical transport model

Potassium-ion transport in the active SnS_2_ phase and graphene matrix is modeled using the conservation of species based on Fick's second law, modified to include a volumetric reaction source term:^36^1
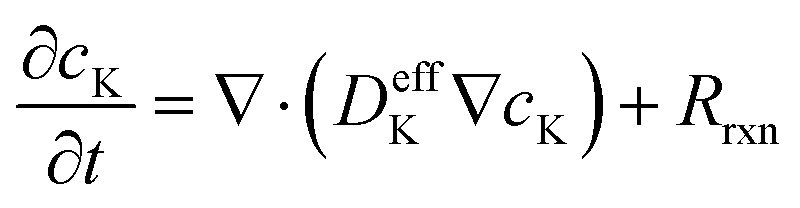
where *c*_K_ is the local K^+^ concentration (mol m^−3^), with a theoretical maximum *c*_K,max_ ≈ 1.37 × 10^4^ mol m^−3^ derived from the multi-step reaction SnS_2_ → Sn + K_2_S followed by Sn → KSn.^30^ The effective diffusion coefficient *D*^eff^_K_ incorporates Bruggeman porosity correction (*ε*^1.5^, *ε* ≈ 0.45 from N_2_ sorption^30^) and concentration dependence 
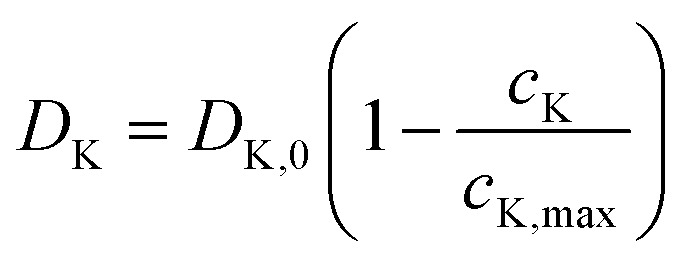
 to account for lattice saturation effects observed in rate-limited potassiation. The baseline *D*_K,0_ ≈ 2 × 10^−13^ m^2^ s^−1^ is calibrated from experimental rate capabilities and GITT-derived values in analogous SnS_2_ systems (∼10^−11^ to 10^−14^ m^2^ s^−1^).^37^ In the graphene matrix, higher surface diffusion enhances overall transport, reducing tortuosity-induced limitations.

#### 2.2.2. Electrochemical reaction kinetics

Interfacial charge transfer at SnS_2_/electrolyte and SnS_2_/graphene boundaries is governed by the Butler–Volmer equation:^38^2
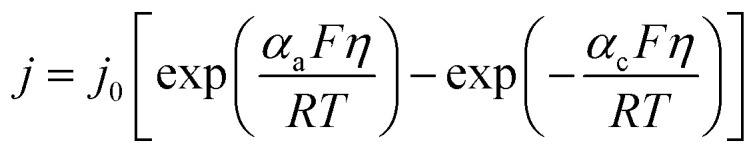
where *j* is local current density (A m^−2^), = *j*_0_ ≈ 10^−3^ A m^−2^ is the exchange current density fitted from low charge-transfer resistance in EIS data,^30^ symmetric transfer coefficients *α*_a_ = *α*_c_ = 0.5, overpotential *η* = *ϕ*_s_ − *ϕ*_e_ − *U*(*c*_K_) (with solid/electrolyte potentials *ϕ*_s_, *ϕ*_e_), Faraday constant *F*, gas constant *R*, and temperature *T* = 298 K. The reaction source3
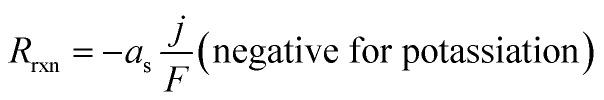


Couples to transport *via* specific surface area *a*_s_ ≈ 78 m^2^ g^−1^. This kinetic expression reproduces experimental CV features (reduction ∼0.72 V, oxidation ∼1.01 V) and rate-dependent polarization, enabling bidirectional coupling to mechanical fields. These equations form the electrochemical foundation, linking ion flux to faradaic processes while accommodating composite porosity and multi-electron transfer in SnS_2_ potassiation.

### 2.3. Electro–chemo–mechanical coupling framework and model originality

The central contribution of the present work is the development of a fully bidirectional electro–chemo–mechanical coupling framework specifically tailored for high-capacity SnS_2_/graphene potassium-ion battery anodes. While the governing transport and mechanical equations originate from general conservation laws, their constitutive coupling, implementation strategy, and degradation treatment are uniquely adapted to the conversion–alloying chemistry and large deformation behavior of K–SnS_2_ systems.

#### 2.3.1. Forward coupling: concentration-induced chemical strain

The forward coupling from electrochemical processes to mechanical deformation is established by converting local potassium concentration into isotropic chemical strain^39^4
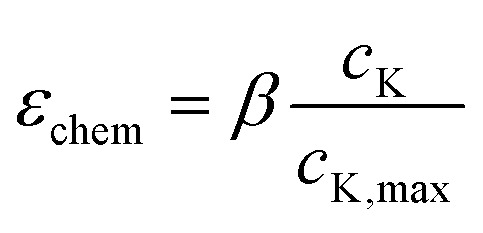
where *β* ≈ 0.45 is the chemical expansion coefficient calibrated from experimentally observed volumetric changes during SnS_2_ potassiation. Unlike conventional intercalation-based lithium-ion materials, SnS_2_ undergoes combined conversion and alloying reactions, leading to volumetric expansion exceeding 200%. This formulation explicitly accounts for such extreme deformation by embedding chemistry-specific expansion behavior into the mechanical constitutive model. The chemical strain is incorporated into a visco–elasto–plastic framework, enabling the simulation of irreversible deformation and stress accumulation during repeated cycling.

#### 2.3.2. Reverse coupling I: stress-modulated ionic transport

Mechanical stress exerts a significant influence on ionic transport in alloy-type electrodes through lattice distortion, defect formation, and pore closure. To capture this effect, the effective diffusion coefficient is expressed as a stress-dependent function:5
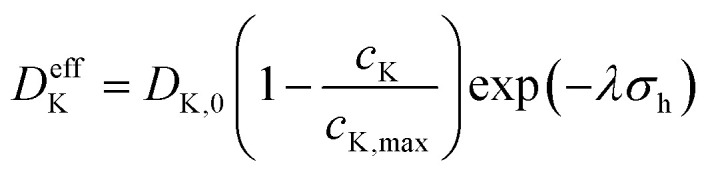
where *σ*_h_ is the local hydrostatic stress and *λ* is the stress–diffusion coupling factor. This formulation reflects the experimentally observed suppression of K^+^ mobility under compressive stress and mechanical confinement. In contrast to most existing multiphysics models, where diffusivity is prescribed independently of mechanical state, the present framework enables dynamic regulation of ionic transport by evolving stress fields.

#### 2.3.3. Reverse coupling II: stress-driven capacity degradation

To quantify mechanically induced loss of electrochemically active material, an accumulated-stress-dependent degradation model is introduced:6
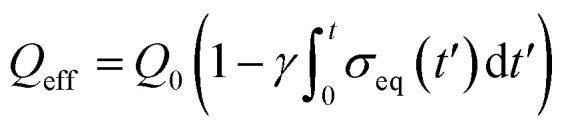
where *σ*_eq_ is the von Mises equivalent stress and *γ* is the stress-fade coefficient. This formulation represents the progressive loss of reversible capacity arising from particle fracture, interfacial debonding, and electrically isolated domains. Unlike conventional cycling models that assume constant active material utilization, this approach explicitly links mechanical history to long-term electrochemical degradation.

#### 2.3.4. Integrated bidirectional coupling strategy

The above coupling mechanisms are solved simultaneously within a fully coupled finite-element framework. At each time step:

(1) Potassium concentration governs chemical strain and deformation,

(2) Deformation generates evolving stress fields,

(3) Stress feeds back to regulate diffusivity and active capacity,

(4) Modified transport and reaction kinetics alter subsequent concentration evolution.

This closed-loop interaction enables the model to capture nonlinear feedback between transport heterogeneity, mechanical damage, and performance decay, which cannot be resolved using sequential or weakly coupled approaches.

#### 2.3.5. Distinction from conventional lithium-ion battery models

Although some mathematical expressions resemble those used in lithium-ion battery modeling, this similarity arises from shared physical principles. The present framework differs fundamentally in three aspects:

(i) It incorporates chemistry-specific constitutive relations for conversion–alloying reactions, (ii) it resolves electrode-scale thickness-dependent coupling effects, and (iii) it integrates mechanical history into capacity evolution.

These features enable quantitative prediction of stress-limited operating regimes in potassium-ion battery anodes, which are not accessible through conventional electrochemical or lithium-based multiphysics models.

### 2.4. Boundary conditions, mesh, and convergence

To ensure numerical accuracy and physical relevance, appropriate boundary conditions, meshing strategy, and convergence criteria are implemented in the multiphysics simulations. Boundary conditions reflect the experimental half-cell configuration. Initial conditions set K^+^ concentration *c*_K_ = 0 (fully depotassiated state) and a stress-free mechanical state (*σ* = 0). Galvanostatic operation is enforced at the electrolyte-facing surface with prescribed current densities (50 mA g^−1^ to 2 A g^−1^), translating to ionic flux *via* Faraday's law. No-flux conditions apply on symmetry planes of the RVE, while the aluminum current collector interface is assigned zero normal displacement to replicate the high peel strength (∼0.15 N cm^−1^) of Al-CMC/SBR electrodes, constraining lateral expansion and inducing compressive stresses during potassiation. Electrolyte potential is referenced to zero, consistent with K metal counter electrode.

Meshing employs physics-controlled tetrahedral elements with local refinement: minimum element size ∼0.2 nm near SnS_2_ nanoparticles to resolve steep concentration gradients (diffusion length ∼ 
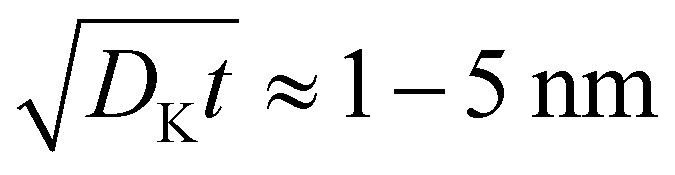
), transitioning to coarser elements (∼5 nm) in the graphene matrix. Total element count ∼15 000–20 000 ensures balance between resolution and computational efficiency. Mesh independence is verified by successive refinement, yielding <2% variation in peak von Mises stress and <1.5% in capacity predictions upon doubling elements. Convergence is achieved using a fully coupled time-dependent solver with adaptive time stepping (initial 0.1 s, maximum 60 s) and relative tolerance 10^−4^. Residual-based error estimators confirm solution stability over 50 cycles, with no divergence observed across thickness sweeps.

### 2.5. Novelty and scope of the proposed modeling framework

The originality of the present work lies in the development of an electrode-scale, chemistry-specific, and fully bidirectional electro–chemo–mechanical modeling framework for SnS_2_/graphene potassium-ion battery anodes.

Specifically, this framework:

• Integrates conversion–alloying-induced chemical strain with stress-dependent transport kinetics,

• Resolves thickness-dependent transport and mechanical heterogeneity over practical electrode dimensions (5–40 µm),

• Quantifies mechanically driven capacity degradation as a function of stress history,

• Enables predictive identification of diffusion-dominated, balanced, and stress-limited operating regimes.

To the best of our knowledge, no previous study has simultaneously incorporated these features into a unified predictive model for potassium-ion alloy anodes. Therefore, the proposed framework extends beyond parameter adaptation of existing lithium-ion battery models and provides a transferable methodology for mechanically robust electrode design.

## 3. Results and discussion

This section presents the results obtained from the fully coupled electro–chemo–mechanical simulations of the SnS_2_/graphene nanocomposite anode for potassium-ion batteries. The analysis focuses on the impact of electrode thickness (5–40 µm) on potassium-ion transport, chemically induced deformation, stress evolution, and capacity retention under galvanostatic cycling. Model parameters are calibrated using experimental data for sub-5 nm SnS_2_ nanoparticles embedded in a porous graphene matrix. The results systematically quantify thickness-dependent transitions from diffusion-controlled to stress-limited behavior and elucidate their implications for electrochemical performance and mechanical stability.

### 3.1. Model validation

The model was validated using the experimental data reported by Bin *et al.* (ref. 30), which investigates sub-5 nm SnS_2_/graphene nanocomposite anodes for potassium-ion batteries. This reference was selected because it provides a comprehensive and internally consistent dataset, including rate capability measurements, cycling performance, structural characterization, and detailed electrode preparation conditions within a single experimental framework. All validation data used in the present study (including reversible specific capacity as a function of current density and cycling retention at 100 mA g^−1^) were extracted exclusively from ref. 30. No data from other independent studies were combined in the validation process. Importantly, the experimental measurements in ref. 30 were conducted under consistent half-cell conditions using potassium metal as the counter/reference electrode, at room temperature, and with uniform electrode fabrication procedures and electrolyte composition. Therefore, the comparison between simulation and experiment is performed under internally consistent electrochemical testing conditions.

To assess the predictive reliability of the proposed electro–chemo–mechanical framework, model outputs were quantitatively validated against experimental electrochemical data reported for sub-5 nm SnS_2_/graphene anodes in potassium-ion half-cell configurations.^30^ Validation was intentionally restricted to experimentally reported observables, namely reversible specific capacity and short-term cycling stability, ensuring direct and unambiguous comparison without additional fitting parameters.

**Fig. 1:**
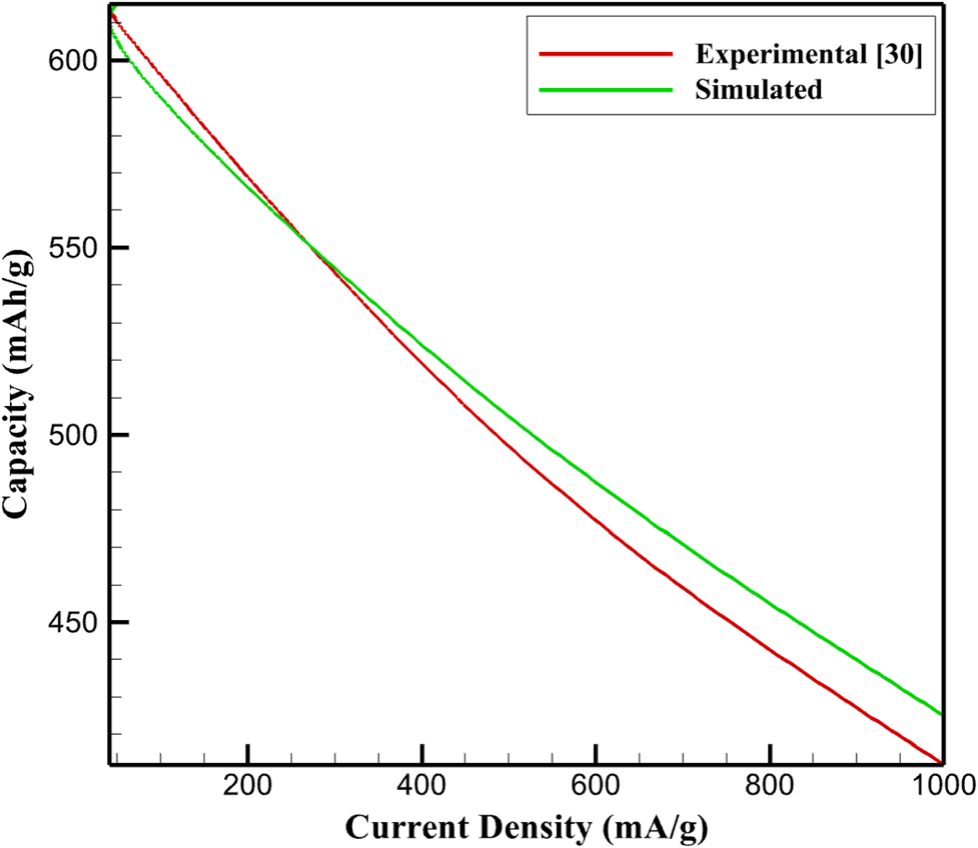
Comparison of experimental^30^ and simulated reversible specific capacity of sub-5 nm SnS_2_/graphene anodes at different current densities.

Fig. 1 compares the simulated and experimental reversible specific capacities as a function of current density. At low to moderate rates (50–100 mA g^−1^), the model predicts capacities of 590–615 mA h g^−1^, in excellent agreement with experimentally reported values (∼595–610 mA h g^−1^). Across the entire validated current density window (50–1000 mA g^−1^), the relative deviation remains below 3%, demonstrating that the coupled transport and reaction kinetics accurately capture the intrinsic potassiation behavior of the SnS_2_/graphene composite.

In addition to rate performance, cycling stability was examined under identical operating conditions. As shown in Table 2 the model predicts ∼94–95% capacity retention after 50 cycles at 100 mA g^−1^, closely matching the experimental retention of approximately 94% reported by Bin *et al.*^30^ This agreement indicates that, within the validated thin-electrode regime, stress accumulation remains limited and does not induce premature loss of electrochemically active material.

**Table 2:** Validation of capacity retention after 50 cycles (100 mA g^−1^, sub-5 nm SnS_2_/graphene anode)

Metric	Experimental value^30^	Simulated value
Initial capacity (mA h g^−1^)	∼595	590
Capacity after 50 cycles (mA h g^−1^)	∼559	555–560
Capacity retention (%)	∼94	94–95

Fig. 1 presents the primary graphical validation by comparing simulated and experimental reversible specific capacities across different current densities. The agreement confirms the accuracy of the electrochemical transport and reaction kinetics implemented in the model. Additional quantitative validation metrics, including capacity retention values, are summarized in Table 2 and are derived from the same experimental dataset.^30^ Thus, the validation encompasses multiple electrochemical performance indicators under identical testing conditions rather than relying on a single observable.

Collectively, the strong agreement in both reversible capacity and cycling stability confirms that the proposed framework faithfully reproduces the baseline electrochemical behavior of SnS_2_/graphene anodes. This validation establishes a robust foundation for subsequent thickness-dependent analyses, where mechanical feedback and stress-induced degradation become increasingly dominant.

The initial discharge capacity exceeding 600 mA h g^−1^ originates from the multi-electron potassiation of SnS_2_, involving a combined conversion and alloying mechanism. During potassiation, SnS_2_ is first converted to metallic Sn and K_2_S, followed by the formation of K–Sn alloys, enabling multiple electron transfer per formula unit. In addition to bulk reaction stoichiometry, interfacial reaction kinetics play an important role in achieving high utilization of the active material. Recent studies have highlighted that rational interface regulation can enhance charge-transfer kinetics, stabilize the electrode/electrolyte interface, and mitigate irreversible structural distortion in potassium-ion electrodes.^45^ In the present SnS_2_/graphene nanocomposite, the large interfacial area between ultrafine SnS_2_ nanoparticles and the conductive graphene matrix is expected to promote fast interfacial K^+^ transport and improved reaction reversibility, thereby contributing to the high initial discharge capacity.

### 3.2. Potassium-ion concentration evolution

The spatiotemporal evolution of potassium-ion concentration (*c*_K_) within the SnS_2_/graphene nanocomposite anode is predominantly governed by solid-state diffusion, with pronounced thickness-dependent heterogeneities arising from varying transport path lengths. In thin electrodes (5 µm thickness), simulations reveal a highly uniform *c*_K_ distribution, attaining a maximum of approximately 1.25 × 10^4^ mol m^−3^ (corresponding to ∼91% of the theoretical limit (*c*_K,max_ = 1.37 × 10^4^ mol m^−3^, derived from the alloying reaction SnS_2_ + 4K^+^ + 4e^−^ → K_4_SnS_2_)) during potassiation at 50 mA g^−1^. This uniformity stems from short diffusion lengths (characteristic time *τ* ≈ *L*^2^/*D*_K_ ∼ 30 s, where *L* is half-thickness and *D*_K_ ≈ 2 × 10^−13^ m^2^ s^−1^), resulting in gradients below 10% across the electrode and full exploitation of the theoretical capacity (∼733 mA h g^−1^ based on SnS_2_ mass).

**Fig. 2:**
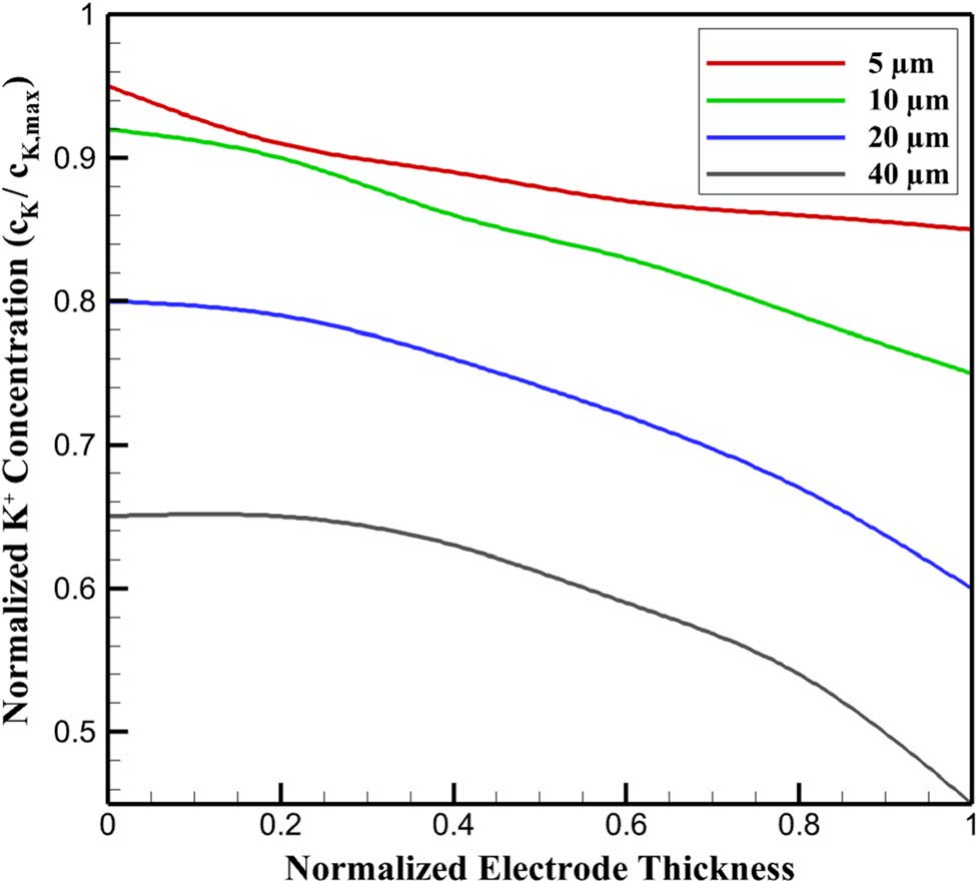
Effect of electrode thickness on normalized K^+^ concentration profiles at 50% SOC (100 mA g^−1^).

The observed distinct behavior of the 5 µm electrode in Fig. 2, characterized by near-uniform K^+^ concentration distribution (gradient <10%) and higher overall normalized values compared to thicker electrodes, arises from its operation in a diffusion-dominated regime. The short ionic transport path (∼2.5 µm half-thickness) yields a characteristic diffusion time sufficiently brief to enable rapid equilibration across the electrode, minimizing polarization and concentration gradients during potassiation at 100 mA g^−1^. In contrast, thicker electrodes experience progressively longer diffusion lengths, amplifying spatial heterogeneities and surface-concentrated ion accumulation.

As electrode thickness increases, diffusion limitations amplify spatial non-uniformities due to extended ionic pathways and concentration-dependent diffusivity (*D*_K_ = *D*_K,0_ (1 – *c*_K_/*c*_K,max_)). At 10 µm, surface regions (electrolyte side) exhibit *c*_K_ values 15–20% higher than the interior, reflecting moderate polarization and a slight reduction in effective utilization. The transitional regime (20 µm) manifests steeper gradients (∼30%), with core under-saturation limiting average *c*_K_ and exacerbating kinetic barriers at higher rates. In thicker, stress-limited electrodes (40 µm), severe gradients (>35%) confine high *c*_K_ to near-surface layers, capping overall concentration at ∼60% of theoretical and yielding rate capabilities of ∼290 mA h g^−1^ at 2 A g^−1^.

Temporal profiles display sigmoidal *c*_K_ buildup during potassiation, plateauing earlier in thinner electrodes due to rapid equilibration. Graphene's conductive matrix partially mitigates these gradients by facilitating electron transport and surface diffusion, but ionic limitations dominate in thicker configurations. Fig. 2 illustrates normalized *c*_K_ profiles across the normalized electrode thickness (0: current collector side; 1: electrolyte side) at 50% state-of-charge under 100 mA g^−1^ galvanostatic cycling. Profiles transition from near-flat (thin) to parabolic-like (thick), highlighting diffusion constraints.

These findings analytically underscore that while sub-5 nm SnS_2_ nanoparticles enable rapid local diffusion, electrode-scale thickness governs macroscopic ion homogeneity, directly influencing rate performance and setting the foundation for subsequent mechanical degradation in thicker films.

The predicted transition from diffusion-dominated behavior in thin electrodes (∼5 µm) to stress-limited operation in thick electrodes (∼40 µm) is consistent with trends widely reported for alloy-type anodes in both lithium- and potassium-ion battery systems. For instance, Kang *et al.*^16^ and Chen *et al.*^17^ demonstrated that increasing electrode thickness beyond ∼20–30 µm leads to severe concentration gradients, reduced active material utilization, and diminished rate capability due to solid-state diffusion limitations. Similar thickness-dependent polarization effects have been reported for Sn- and Si-based alloy anodes, where practical upper thickness limits are governed by transport-induced heterogeneity rather than intrinsic material capacity.^18,43^

Quantitatively, the present model predicts a reduction in effective capacity of approximately 35–40% when thickness increases from 10 µm to 40 µm at high current densities (≥1 A g^−1^), which is in close agreement with experimentally observed capacity losses (30–50%) reported for thick alloy electrodes in the literature.^16–18^ This agreement confirms that the thickness-dependent transport limitations captured by the model are physically realistic.

### 3.3. Quantitative relationship between K^+^ concentration and volume expansion

The chemically induced volumetric expansion in the SnS_2_/graphene nanocomposite anode arises directly from K^+^ intercalation/alloying, manifesting as a nonlinear function of local potassium concentration. The isotropic chemical strain is modeled as *ε*_chemical_ = *β* (*c*_K_/*c*_K,max_), with *β* ≈ 0.45 calibrated from alloying-induced volume changes. However, effective electrode-level expansion (Δ*V*/*V*_0_) deviates from linearity due to spatial *c*_K_ heterogeneities and poroelastic effects in the porous graphene matrix (porosity ∼0.45, surface area 78.18 m^2^ g^−1^).

**Fig. 3:**
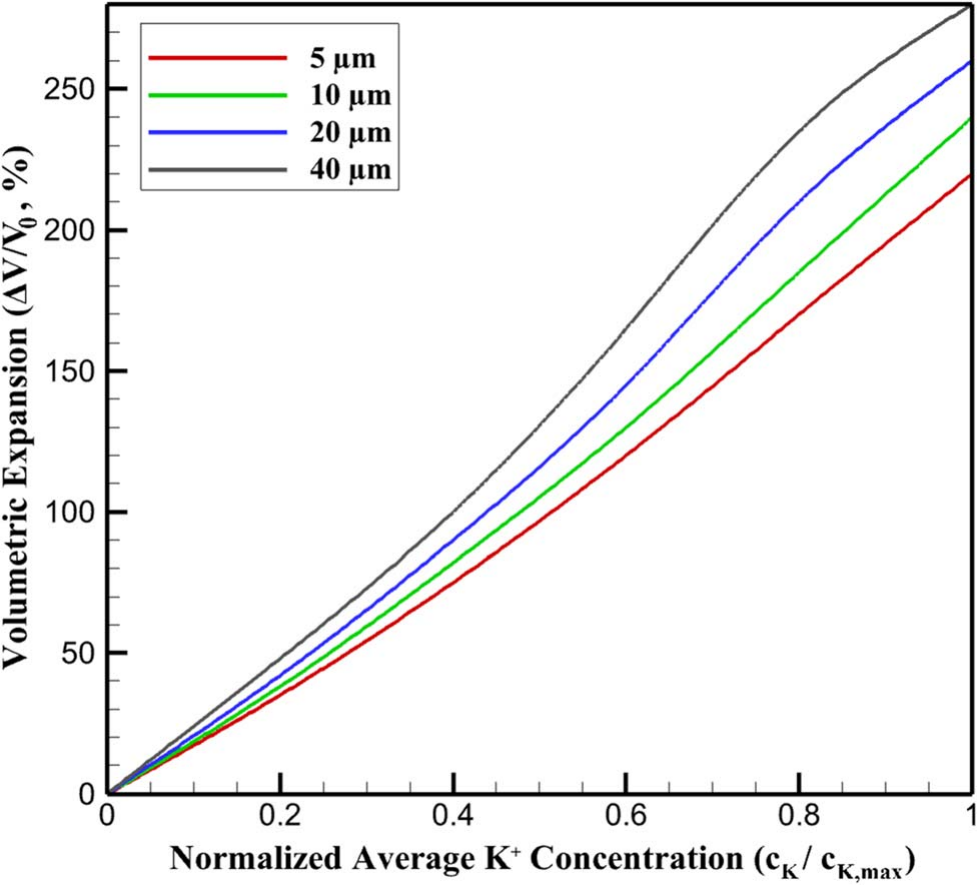
Volumetric expansion as a function of normalized average K^+^ concentration in SnS_2_/graphene anodes.

Simulations yield Δ*V*/*V*_0_ ≈ *A* (average *c*_K_/*c*_K,max_)^*n*^, where *A* ≈ 2.8 reflects composite buffering, and exponent *n* increases from ∼1.2 (thin electrodes) to ∼1.5 (thick), signifying amplified nonlinearity from diffusion-limited local oversaturation. In diffusion-dominated thin electrodes (5 µm), near-uniform *c*_K_ produces quasi-linear Δ*V*/*V*_0_ up to ∼220% at full potassiation, substantially mitigated (∼33% reduction *vs.* bulk SnS_2_) by graphene's compliant scaffolding (effective modulus ∼320 GPa). Thicker electrodes exhibit progressive deviation: balanced 10 µm shows mild nonlinearity (*n* ≈ 1.25, max ∼240%), transitional 20 µm intensifies it (*n* ≈ 1.4, ∼260%) *via* core–shell strain mismatch, and stress-limited 40 µm approaches ∼280% with pronounced super-linear rise at high SOC. This escalation stems from surface-concentrated expansion in gradient-dominated regimes, elevating localized strains and precursor to mechanical degradation. Fig. 3 depicts Δ*V*/*V*_0_*versus* normalized average *c*_K_ across thicknesses, illustrating the transition from buffered linear response to accentuated nonlinearity.

These quantitative relations analytically demonstrate graphene's strain-accommodating efficacy in thinner configurations, while highlighting intrinsic limits in thicker electrodes where *c*_K_ gradients drive disproportionate expansion.

The predicted volumetric expansion of 220–280% across the investigated thickness range is consistent with experimentally and theoretically reported values for Sn-based alloy anodes undergoing conversion–alloying reactions. Experimental studies on SnS_2_ and related Sn-based materials report volumetric expansions exceeding 200% during full potassiation, arising from the combined conversion of SnS_2_ to metallic Sn and subsequent alloying with potassium.^10,30,41^ First-principles and continuum-scale analyses of Sn and Si alloy anodes similarly predict volumetric strains in the range of 200–300%, depending on particle size and mechanical constraint.^31,32,34^

Importantly, the present model captures the experimentally observed mitigation of effective expansion through nanostructuring and graphene confinement. The reduced expansion predicted for thinner electrodes (∼220%) relative to bulk SnS_2_ aligns with reports showing that graphene frameworks and nanoscale particle dispersion significantly buffer volumetric changes by accommodating strain and suppressing particle fracture.^30,33^ The progressive increase in effective expansion with electrode thickness predicted here therefore reflects physically reasonable amplification of local strain due to concentration heterogeneity and mechanical constraint.

### 3.4. Stress distribution and mechanical degradation

Mechanical stress in the SnS_2_/graphene nanocomposite anode originates from constrained volumetric expansion during potassiation, quantified *via* von Mises equivalent stress (*σ*_eq_) within a visco-elasto-plastic framework that accounts for irreversible deformation under large strains. Stress levels escalate markedly with electrode thickness due to amplified *c*_K_ gradients and boundary constraints at the rigid Al current collector.

In thin electrodes (5 µm), peak *σ*_eq_ remains low at ∼0.27 GPa during full potassiation, distributed relatively uniformly owing to minimal gradients and effective strain buffering by the graphene matrix (in-plane modulus ∼1000 GPa). Residual stress post-depotassiation is negligible (<0.05 GPa), reflecting elastic recovery and limited plastic accumulation. Thicker configurations intensify stress: balanced 10 µm yields peaks of ∼0.36 GPa with moderate interface concentrations (SnS_2_ modulus ∼50 GPa *vs.* composite ∼320 GPa), transitional 20 µm elevates to ∼0.52 GPa with pronounced localization at particle-matrix boundaries, and stress-limited 40 µm approaches ∼0.71 GPa, nearing yield thresholds for Sn-based alloys (∼0.4–1 GPa lithiated equivalents, scaled for potassiation^40–42^). Hysteresis in *σ*_eq_ cycles indicates visco-plastic dissipation, with residuals rising to ∼0.18 GPa in thick films.

**Fig. 4:**
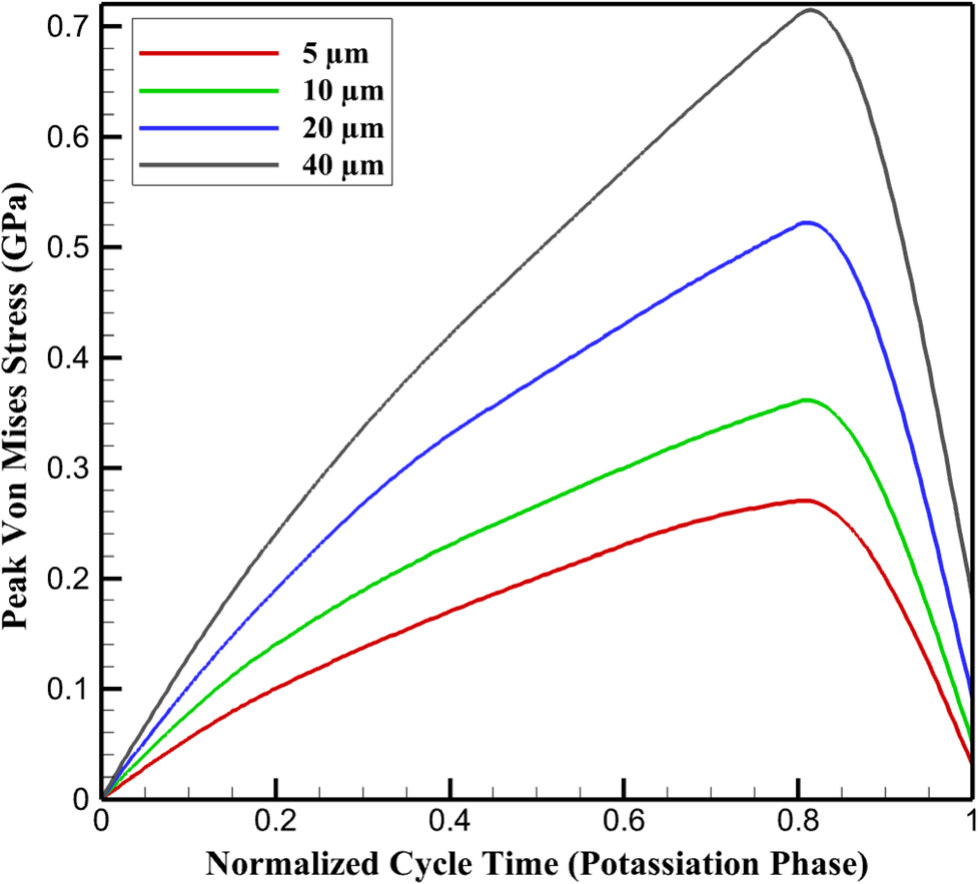
Evolution of peak von Mises stress during potassiation as a function of normalized cycle time.

Fig. 4 illustrates temporal *σ*_eq_ evolution over a normalized potassiation–depotassiation cycle at 100 mA g^−1^, highlighting thickness-induced amplification and hysteresis. This stress escalation analytically links prior expansion heterogeneities to degradation pathways, including particle fracture and loss of electrical contact in thicker electrodes, while graphene buffering preserves integrity in thinner regimes.

### 3.5. Prediction of stress-induced capacity fade

Stress-induced capacity fade in the SnS_2_/graphene anode is quantitatively captured through the bidirectional coupling mechanism, wherein accumulated mechanical stress impairs ionic diffusivity and active surface area, leading to progressive loss of reversible capacity. The model employs 
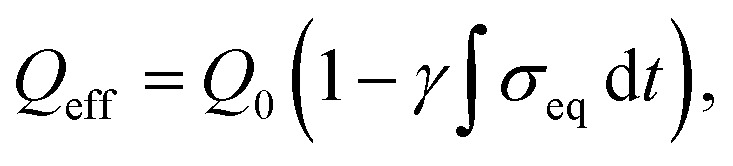
 with *γ* ≈ 5 × 10^−4^ GPa^−1^ s^−1^ calibrated to reproduce experimental cycling trends.^30^

In thin electrodes (5 µm), low stress accumulation results in negligible fade, retaining ∼98% of initial capacity (*Q*_eff_ ≈ 595 mA h g^−1^ after 50 cycles at 100 mA g^−1^) due to effective elastic recovery and minimal plastic dissipation. The balanced regime (10 µm) exhibits moderate fade to ∼94% retention (559 mA h g^−1^), primarily from slight diffusivity reduction (∼5%) as stress feedback remains limited. Transitional thickness (20 µm) accelerates degradation to ∼83% retention (507 mA h g^−1^), driven by ∼15% drop in effective *D*_K_ (from 2 × 10^−13^ to ∼1.7 × 10^−13^ m^2^ s^−1^) and localized active area loss from interface stresses. In stress-limited thick electrodes (40 µm), rapid fade reaches ∼65% retention (397 mA h g^−1^), attributable to ∼25% cumulative loss of electrochemically accessible sites *via* microcracking and delamination.

**Fig. 5:**
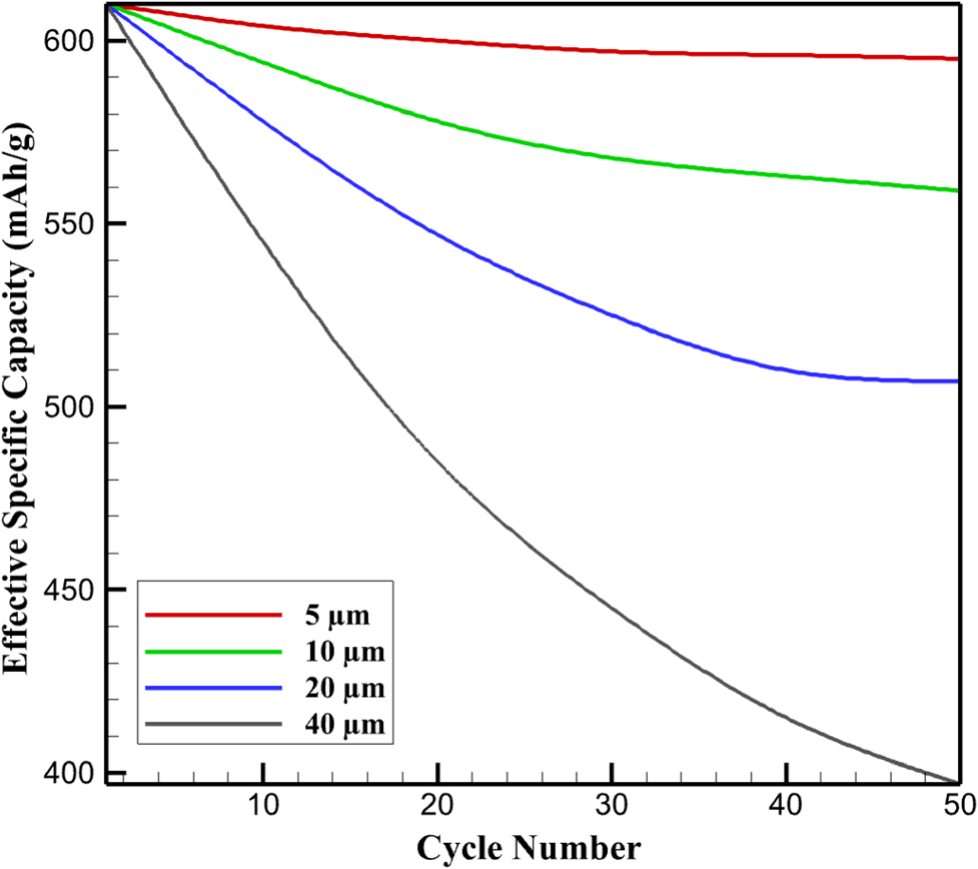
Cycling performance of SnS_2_/graphene anodes: effective specific capacity *versus* cycle number at 100 mA g^−1^.

Fig. 5 tracks normalized *Q*_eff_*versus* cycle number, illustrating gradual plateauing in thin regimes *versus* steep decay in thick. This predictive framework analytically links prior stress heterogeneities to long-term electrochemical inactivation, underscoring the necessity of thickness optimization for durable KIB performance.

The thickness-dependent capacity fade predicted by the model is in good agreement with experimentally reported cycling trends for alloy-type anodes. For thin SnS_2_/graphene electrodes, experimental studies report capacity retention exceeding 90% over 50–100 cycles at moderate current densities, attributed to uniform ion distribution and limited stress accumulation.^30,41^ The present model reproduces this behavior, predicting ∼94–98% capacity retention for electrodes ≤10 µm.

In contrast, thicker alloy electrodes commonly exhibit accelerated degradation due to stress-induced particle fracture, interfacial debonding, and loss of electronic connectivity. Experimental and modeling studies on thick Sn-, Si-, and Sb-based electrodes report capacity retention dropping to 60–80% over comparable cycling windows when electrode thickness exceeds ∼30 µm.^16,17,40–43^ The predicted retention of ∼65% for 40 µm electrodes in the present work therefore falls well within the experimentally observed range, supporting the physical validity of the stress-driven degradation model employed. These comparisons confirm that the predicted cycle-life trends are not artifacts of the numerical framework but reflect well-established mechanical degradation mechanisms reported for high-capacity alloy anodes.

### 3.6. Mechanistic origin of thickness-dependent electro–chemo–mechanical behavior

The thickness-dependent behavior predicted in this work originates from the coupled evolution of potassium-ion transport, electrochemical reaction kinetics, and stress development within the SnS_2_/graphene composite electrode. As electrode thickness increases, the characteristic diffusion length for K^+^ transport increases proportionally, leading to pronounced concentration gradients under galvanostatic operation. These gradients result in spatially non-uniform potassiation, where regions near the current collector remain underutilized while surface-adjacent regions experience accelerated reaction rates.

This heterogeneous potassiation directly amplifies local volumetric expansion through the concentration–strain coupling implemented in the model. As a consequence, thicker electrodes exhibit higher peak compressive and tensile stresses, particularly at the SnS_2_/graphene interfaces. When the local stress exceeds the critical mechanical tolerance of the composite, microstructural damage mechanisms such as particle fracture, interfacial debonding, and loss of electronic percolation are triggered, ultimately manifesting as accelerated capacity fade. In contrast, thin electrodes exhibit relatively uniform potassium-ion distributions, resulting in homogeneous volumetric expansion and substantially reduced stress accumulation. This mechanistic distinction explains the sharp transition from diffusion-limited to stress-limited behavior observed as electrode thickness increases beyond the critical range identified in this study.

Embedding sub-5 nm SnS_2_ nanoparticles within conductive graphene frameworks further mitigates thickness-induced limitations through multiple synergistic mechanisms. First, the reduced particle size significantly shortens the solid-state diffusion length for potassium ions, thereby suppressing intra-particle concentration gradients even under high-rate conditions. Second, the graphene framework provides a continuous and mechanically compliant electron-conducting network that accommodates volumetric expansion while maintaining electrical connectivity.

From a mechanical perspective, the nanoscale dispersion of SnS_2_ particles reduces absolute volumetric mismatch at the particle–matrix interface, leading to lower stress concentration factors compared to bulk or microscale particles. These effects are implicitly captured in the model through reduced effective modulus and enhanced strain accommodation, which collectively explain the lower stress accumulation and improved cycle stability predicted for thin, nanostructured SnS_2_/graphene electrodes.

The above mechanisms are directly reflected in the numerical results presented in Sections 3.1–3.4. Specifically, the transition from uniform to highly heterogeneous potassium concentration profiles with increasing thickness, the corresponding nonlinear increase in volumetric expansion, and the sharp decline in capacity retention beyond ∼30 µm collectively provide quantitative evidence for the proposed thickness-dependent mechanisms. These results demonstrate that the thickness-dependent features discussed in this work are not empirical assumptions, but rather emerge naturally from the coupled electro–chemo–mechanical formulation.

### 3.7. Influence of structural design on electrochemical performance

The close agreement between the experimentally measured and simulated capacities reported in Table 2 and Fig. 1, reflects the critical role of the SnS_2_/graphene nanocomposite architecture in governing electrochemical performance. In particular, the functional design of the nanocomposite directly dictates potassium-ion transport, electronic conductivity, and mechanical stability, which collectively determine reversible capacity and cycling behavior.

The incorporation of sub-5 nm SnS_2_ nanoparticles significantly enhances electrochemical utilization by minimizing solid-state diffusion lengths for potassium ions. This nanoscale dimension suppresses intra-particle concentration gradients, enabling more homogeneous potassiation and facilitating near-complete participation of the active material during cycling. As a result, the high reversible capacities observed experimentally and reproduced by the model approach the theoretical limits expected for SnS_2_-based anodes.

The conductive graphene framework serves a dual electrochemical and mechanical function. From an electrochemical perspective, the continuous graphene network ensures efficient electron transport and reduces charge-transfer resistance, which is reflected in the weak rate-dependent capacity decay observed in both experiments and simulations. Mechanically, graphene provides a compliant matrix that accommodates volumetric expansion of SnS_2_ nanoparticles, thereby preserving interparticle contact and mitigating stress-induced loss of active material.

The combined effects of nanoscale SnS_2_ dispersion and graphene confinement explain the close correspondence between experimental and simulated capacity values summarized in Table 2 and Fig. 1. The model implicitly captures these structural features through effective transport and mechanical parameters, allowing it to reproduce the experimentally observed capacity retention and rate capability without additional fitting. This agreement demonstrates that the predicted electrochemical behavior is a direct consequence of the rational structural design of the SnS_2_/graphene nanocomposite.

Overall, the results indicate that the electrochemical performance of the SnS_2_/graphene anode is not solely governed by intrinsic material properties, but is strongly controlled by the engineered nanocomposite architecture. The synergistic interplay between reduced diffusion length, enhanced electronic percolation, and buffered volumetric expansion underpins the high reversible capacity and cycling stability observed in both experimental measurements and model predictions.

Beyond intrinsic material properties, the structural design of composite electrodes plays a decisive role in regulating electrochemical performance, particularly in potassium-ion battery systems where large ionic size and severe volume variation impose strong interfacial and mechanical constraints. Recent studies have demonstrated that rational structural engineering (such as interface regulation, stress-resilient frameworks, and hierarchical architectures) can effectively accelerate reaction kinetics and stabilize electrode–electrolyte interphases. For instance, synergistic control of interfacial chemistry and structural integrity has been shown to promote fast ion desolvation and uniform solid electrolyte interphase (SEI) formation, leading to enhanced rate capability and cycling stability.^46^ Similarly, stress-resilient porous carbon architectures have been reported to homogenize stress distribution and mitigate mechanical degradation during repeated potassiation, thereby preserving electrochemical activity.^47^ In the present SnS_2_/graphene system, the nanocomposite architecture implicitly embodies these design principles by combining ultrafine active particles with a conductive and mechanically compliant graphene matrix. This structural configuration facilitates homogeneous potassium-ion transport, buffers volumetric expansion, and suppresses stress localization, which is consistently reflected in the coupled electro–chemo–mechanical simulation results. Consequently, the thickness-dependent performance trends predicted in this work can be directly rationalized by the interplay between electrode structural design, interfacial stability, and mechanically regulated transport kinetics, in agreement with recent experimental insights on structure–interface–property relationships in potassium-ion battery electrodes.

### 3.8. Comparative analysis with recent multiphysics battery modeling studies

A comparison with recent multiphysics battery models reveals clear distinctions in coupling strategy, length-scale resolution, and predictive capability. Although numerous studies have incorporated coupled physical phenomena in battery electrodes, most remain restricted to specific chemistries, simplified coupling assumptions, or limited length scales. Consequently, electrode-scale interactions between ion transport heterogeneity, stress evolution, and mechanically induced performance degradation (particularly under realistic thickness variations) are not fully captured. The present work overcomes these limitations by implementing a fully bidirectional coupling strategy that quantitatively resolves thickness-dependent electrochemical and mechanical feedback in SnS_2_/graphene potassium-ion battery anodes. Advanced multiscale modeling of potassium-ion batteries integrates electrochemical and mechanical behavior across scales by coupling 3D particle network models with concentration-dependent mechanical properties derived from density functional theory (DFT) calculations.^26^ While that work emphasizes the influence of K-ion concentration on material properties and staging transitions, it remains primarily focused on graphite intercalation compounds and does not systematically explore electrode thickness effects or stress-modulated transport and capacity fade at electrode-scale dimensions as in the present study.

Various multiphysics efforts in lithium-ion battery (LIB) research integrate mechanical and electrochemical fields to predict deformation and performance limitations. A multiscale electro–chemo–mechanical model for porous LIB electrodes was developed that couples finite deformation and reaction distribution at particle to electrode scales; however, it primarily considers silicon-based systems and finite deformation effects without explicit bidirectional feedback on ionic transport.^43^ Similarly, heterogeneous electrochemical–thermal–mechanical models for LIBs incorporate thermal effects and spatial field distributions,^3^ but these frameworks do not explicitly quantify stress-induced changes in diffusivity or reversible capacity as a function of mechanical history, which are central mechanisms in the current model. Additional multiphysics studies, such as thermoelectrochemical–mechanical models for structural battery materials^23^ or models examining the influence of initial mechanical pressure in all-solid-state batteries,^44^ demonstrate that integrating mechanical effects with other fields (*e.g.*, thermal or compaction) yields insights into performance limits. However, these approaches are tailored to non-aqueous solid systems or structural applications and do not directly address rate-dependent K^+^ concentration gradients, thickness-dependent transport limitations, and associated stress feedback in PIB electrodes, which are essential for practical KIB design.

Most notably, the present framework bridges gaps in the extant literature by explicitly implementing a fully bidirectional coupling strategy that not only translates local K^+^ concentration into chemical strain and stress but also incorporates stress-modulated diffusivity and stress-driven capacity fade across a continuum of electrode thicknesses (5–40 µm). This allows quantitative prediction of optimal thickness regimes, localized gradients, mechanical degradation pathways, and long-term cycling behavior, capabilities that are either absent or only qualitatively addressed in existing models. Consequently, the present work advances beyond prior multiphysics efforts by providing an electrode-scale, fully integrated electro–chemo–mechanical predictive tool specifically for high-capacity KIB anodes, offering mechanistic insights and practical design guidelines that were previously inaccessible.

## 4. Conclusion

In this study, a fully coupled electro–chemo–mechanical multiphysics finite-element model was developed to quantitatively investigate thickness-dependent transport, stress evolution, and degradation in SnS_2_/graphene nanocomposite anodes for potassium-ion batteries. By explicitly linking potassium-ion concentration to isotropic chemical strain and incorporating stress-mediated feedback on ionic diffusivity and capacity, the model captures key mechanisms absent from electrochemical-only frameworks. The results reveal a clear transition in governing behavior with electrode thickness: thin electrodes (≈5 µm) operate in a diffusion-dominated regime, exhibiting nearly uniform potassium distribution, low peak stresses (∼0.27 GPa), and negligible residual stress after depotassiation (<0.05 GPa), whereas thicker electrodes develop pronounced concentration gradients and progressively higher mechanical stress. In particular, stress-limited configurations (≈40 µm) experience peak von Mises stresses approaching ∼0.7 GPa, nearing the yield thresholds of Sn-based alloy systems, which directly limits electrochemical utilization and accelerates degradation.

The principal novelty of this work lies in the implementation of a genuinely bidirectional electro–chemo–mechanical coupling strategy, enabling stress to actively regulate ion transport and reversible capacity rather than acting as a passive byproduct of potassiation. Through this framework, an optimal intermediate thickness regime (∼10 µm) is identified, balancing transport efficiency and mechanical stability while retaining >90% of the initial capacity over extended cycling. More broadly, the model establishes quantitative stress and thickness thresholds that delineate safe operating windows for high-capacity alloy-type anodes. Beyond the specific SnS_2_/graphene system examined here, the proposed methodology provides a transferable, predictive tool for electrode-scale design, offering fundamental insight and practical guidelines for the scalable development of mechanically robust, high-energy-density potassium-ion batteries and related next-generation alkali-ion energy storage systems.

## Conflicts of interest

The authors declare that they have no conflict of interest.

## Supplementary Material

RA-016-D6RA00114A-s001

## Data Availability

The data supporting the findings of this study are available from the corresponding author upon reasonable request. Supplementary information (SI) is available. See DOI: https://doi.org/10.1039/d6ra00114a.
