# Supplementary material for: Thickness-dependent electro–chemo–mechanical modelling of SnS2/graphene anodes for high-performance potassium-ion batteries
Source: RSC Adv. 2026 Mar 19;16(17):15510–22. doi: 10.1039/d6ra00114a (PMC13000893; doi:10.1039/d6ra00114a)
Supplement: RA-016-D6RA00114A-s001 [file RA-016-D6RA00114A-s001.pdf]

Supplementary Information (SI) for RSC Advances.
This journal is © The Royal Society of Chemistry 2026

Computational Methods and Governing Equations

To ensure transparency and reproducibility of the proposed electro–chemo–mechanical framework, the theoretical models, governing equations, and parameter sources employed in the simulations are summarized here. The model is implemented in COMSOL Multiphysics 6.2 by coupling ion transport, electrochemical kinetics, and solid mechanics within a finite-element formulation.

1. Ion Transport

Potassium-ion transport in the SnS_2/graphene composite is described by mass conservation with a reaction source term:

$$\frac{\partial c_K}{\partial t} = \nabla \cdot (D_K \nabla c_K) + \frac{R}{F}$$

Where c_K is the K^+ concentration (mol m^{-3}), D_K is the effective diffusion coefficient, R is the volumetric reaction rate, and F is Faraday's constant.

The diffusion coefficient includes porosity and stress effects:

$$D_K = D_{K,0}\varepsilon^{1.5}(1 - \frac{c_K}{c_{K,max}})exp(-\lambda\sigma_h)$$

Where $D_{K,0}$ is the intrinsic diffusivity, ε is electrode porosity, $c_{K,max}$ is the maximum potassium concentration, λ is the stress–diffusion coupling coefficient, and σ_h is hydrostatic stress. This formulation follows stress-assisted diffusion concepts commonly used for alloy electrodes.

The theoretical maximum concentration $c_{K,max}$ f0 $\approx 1.37\times10^4$ mol m^{-3} is derived from the conversion–alloying reaction of SnS_2 with potassium.

2. Electrochemical Kinetics

The interfacial reaction kinetics are governed by the Butler–Volmer equation:

$$j = j_0[exp(\frac{\alpha_a F\eta}{RT}) - exp(-\frac{\alpha_c F\eta}{RT})]$$

Where j is local current density, j_0 is the exchange current density, $\alpha_a=\alpha_c=0.5$ are transfer coefficients, R is the gas constant, T is temperature, and $\eta=\phi s-\phi e-U(c_K)$ is the overpotential.

The volumetric source term coupled to transport is:

$R = a_s j$

Where a_s is the specific surface area of the composite electrode. Parameter values are taken from experimental SnS_2/graphene electrodes and calibrated against capacity data.

3. Mechanical Model and Chemical Strain

Mechanical deformation is described using linear elasticity with concentration-induced chemical strain:

$$\varepsilon = \varepsilon^{el} + \varepsilon^{ch}$$

$$\varepsilon^{ch} = \beta c_{K,max} c_K I$$

Where β is the chemical expansion coefficient and I is the identity tensor. This approach follows classical chemo-mechanical formulations for alloy anodes.

The stress tensor is obtained from:

$$\sigma = C : (\varepsilon - \varepsilon^{ch})$$

With C being the elastic stiffness tensor derived from the effective Young's modulus of the SnS_2/graphene composite.

4. Stress-Driven Capacity Degradation

Mechanical degradation is introduced through stress-dependent loss of active material:

$$\frac{dQ_{eff}}{dt} = -\gamma \sigma_{eq} Q_{eff}$$

Where Q_{eff} is effective capacity, γ is the stress-fade coefficient, and σ_{eq} is the von Mises stress. Similar formulations have been used to link mechanical damage to electrochemical performance in multiphysics battery models.

5. Simulated Parameters and References

Key simulated values and their sources are summarized below:

Parameter	Value	Source
$D_{K,0}$	(2×10^{-13}) m² s⁻¹	Zhang et al. [37]
Porosity (ε)	0.45	Bin et al. [30]
$c_{K,max}$	(1.37×10^{4}) mol m⁻³	Bin et al. [30]
j_0	(10^{-3}) A m⁻²	Bin et al. [30]

Chemical expansion (β)	0.45	Christensen & Newman [31], Zhao et al. [32]
Young's modulus (E)	320 GPa	Zhao et al. [32], Bucci et al. [33]
Stress–diffusion coupling (λ)	(1×10^{-9}) Pa^{-1}	Luo et al. [39]
Stress-fade coefficient (γ)	(5×10^{-4}) GPa^{-1} s^{-1}	Bin et al. [30] (calibrated)

All parameters were extracted from the literature on SnS_2/graphene and alloy-type anodes or calibrated against experimental data. Diffusion coefficients were taken from Zhang et al. [37]. Structural and electrochemical parameters were obtained from Bin et al. [30]. Chemo-mechanical properties were adopted from classical alloy anode models by Christensen and Newman [31], Zhao et al. [32], and Bucci et al. [33]. Stress-coupling parameters follow the formulation of Luo et al. [39].
